# Supplementary material for: Fetal biparietal diameter as a potential risk factor for prolonged second stage of labor: A retrospective observational cohort study
Source: PLoS One. 2020 Oct 1;15(10):e0239045. doi: 10.1371/journal.pone.0239045 (PMC7529230; doi:10.1371/journal.pone.0239045)
Supplement: Attachment — Submitted filename: Response to Reviewers.docx [file pone.0239045.s004.docx]

August 08, 2020

Frank T. Spradley

Academic Editor

*PLOS ONE*

Dear Editor:

I, along with my co-authors, would like to re-submit the attached manuscript, titled “Fetal biparietal diameter as a potential risk factor for prolonged second stage of labor: a retrospective observational cohort study” as an original research article (manuscript ID: PONE-D- 20-13066).

We thank you and the reviewers for your thoughtful suggestions and insights, which have been very useful to us in improving the quality of our manuscript. All changes are shown in red in the revised manuscript. Our point-by-point responses to all reviewer comments have been prepared and provided below.

We believe that the changes made based on reviewer comments have significantly improved our manuscript, and we hope that you will find it suitable for publication in *PLOS ONE*.

Thank you for your consideration. I look forward to hearing from you.

Sincerely,

Dr. Satoshi Shinohara

Department of Obstetrics and Gynecology

National Hospital Organization Kofu National Hospital

11-35 Tenjin, Kofu, Yamanashi 400-8533, Japan

Tel: +81-55-253-6131

Fax: +81-55-251-5597

Email: shinohara617@gmail.com

**RESPONSE TO REVIEWER 2**

**Comment 1**

incidence refers to the proportion or rate of persons who develop a condition during a particular time period. From the best of my understanding here in your data there is time to event data(starting from the onset of labour-----Time of delivery ) "Prolonged labour" by it self deals about time. Therefore, please think twice about your data and model selection . Even Just after you compete logistic regression Odds ratio is not appropriate measuring association for cohort study rather risk ratio sounds good.

**Response 1**

Thank you for your comment. We have carefully reconsidered the analysis method based on your suggestion. As stated in your comment, we think the Cox proportional hazard model is appropriate if we treat the second stage of labor as time to event data. However, in this study we focused on only the event of the prolonged second stage of labor. Similar to our study, there are several studies that have conducted logistic analyses focusing on only the event of the prolonged second stage of labor (1~2):

1. Lina Salman, Anat Shmueli, Amir Aviram, et al. The association between neonatal head circumference and second stage duration.: J Matern Fetal Neonatal Med. 2019 Dec;32(24):4086-4092.
2. Eyal Sheiner, Asnat Walfisch, Mordechai Hallak, et al. Length of the second stage of labor as a predictor of perineal outcome after vaginal delivery.: J Reprod Med. 2006 Feb;51(2):115-119.

Moreover, we rechecked the references (3-9 listed below) cited in our paper. According to these studies, there are only reports that have compared maternal and fetal morbidities between prolonged second stage labor groups and non-prolonged second stage of labor groups, and there is no study that has examined the relation between maternal and fetal morbidities with prolonged second stage of labor as a continuous variable. To date, there is insufficient evidence as to whether a longer the second stage of labor corresponds with a poor perinatal prognosis. Rather, it can be understood that if the second stage of labor is not delayed, it does not matter in the clinical setting. Therefore, we consider a logistic analysis to be the correct examination method in this study.

3. Le Ray C, Fraser W, Rozenberg P, Langer B, Subtil D, Goffinet F, et al. Duration of passive and active phases of the second stage of labour and risk of severe postpartum haemorrhage in low-risk nulliparous women. Eur J Obstet Gynecol Reprod Biol 2011;158: 167–172.
4. Stephansson O, Sandström A, Petersson G, Wikström AK, Cnattingius S. Prolonged second stage of labour, maternal infectious disease, urinary retention and other complications in the early postpartum period. BJOG 2016;123: 608–616.
5. Cheng YW, Hopkins LM, Laros RK Jr, Caughey AB. Duration of the second stage of labor in multiparous women: maternal and neonatal outcomes. Am J Obstet Gynecol 2007;196: 585.e1–6.
6. Allen VM, Baskett TF, O'Connell CM, McKeen D, Allen AC. Maternal and perinatal outcomes with increasing duration of the second stage of labor. Obstet Gynecol 2009;113: 1248–1258.
7. Quiñones JN, Gómez D, Hoffman MK, Anath CV, Smulian JC, Skupski DW, et al. Length of the second stage of labor and preterm delivery risk in the subsequent pregnancy. Am J Obstet Gynecol 2018;219: 467.e1–467.e8.
8. Laughon SK, Berghella V, Reddy UM, Sundaram R, Lu Z, Hoffman MK. Neonatal and maternal outcomes with prolonged second stage of labor. Obstet Gynecol 2014;124: 57–67.
9. Sandström A, Altman M, Cnattingius S, Johansson S, Ahlberg M, Stephansson O. Durations of second stage of labor and pushing, and adverse neonatal outcomes: a population-based cohort study. J Perinatol 2017;37: 236–242.

**Comment 2**

In the abstract, line 38- 42. This is hard to read. It needs clarification.

**Response 2**

Thank you for your comment. According to your comment, we rewrote the relevant part as, “Kaplan-Meier survival analysis showed that at each time point during the second stage of labor, the percentage of women who had not yet delivered was higher among those who delivered neonates with large BPD Z-scores than among those who delivered neonates with smaller BPD Z-scores. ”.

**Comment 3**

Line 42, in the contrary, in multiparous women…., there was no mention for nulliparous women at all.

**Response 3**

Thank you for your comment. However, our data regarding nulliparous women were mentioned in the Abstract as follows: “In nulliparous women, multivariable analysis indicated that BPD Z-score was significantly associated with prolonged second stage of labor (adjusted odds ratio, 1.18; 95% confidence interval, 1.02–1.37).”.

**Comment 4**

Line 134, To identify the potential indicators of prolonged second stage of labor… could be removed and start the sentence with prolonged second stage of labor was defined as……

**Response 4**

Thank you for your comment. According to your suggestion, we removed “To identify the potential indicators of prolonged second stage of labor,” and replaced it with, “Prolonged second stage of labor was defined as…”.

**Comment 5**

Table 2 would be subdivided into nulliparous and multiparous women so that tables 3 and 6 would be presented in one table (table 2).

**Response 5**

Thank you for your comment. Table 2 includes the baseline characteristics of the study population. We considered this information to be important for understanding the whole study population and have not changed it. We have, instead, added fourth-degree perineal lacerations, Ap score (5 min), and UA pH to Table 3 and Table 6 as well as Table 2.

**Comment 6**

line 334, Nulliparity should be replaced by nulliparous.

**Response 6**

Thank you for your comment. According to your suggestion, we changed the wording from nulliparity to nulliparous (page 27, line 331).
