# Supplementary material for: Fetal biparietal diameter as a potential risk factor for prolonged second stage of labor: A retrospective observational cohort study
Source: PLoS One. 2020 Oct 1;15(10):e0239045. doi: 10.1371/journal.pone.0239045 (PMC7529230; doi:10.1371/journal.pone.0239045)
Supplement: Attachment — Submitted filename: reviewer comment.docx [file pone.0239045.s001.docx]

Dear editor,

Many thanks for giving me this opportunity to review this attractive article. I look forward for further contribution to your imminent journal. Kindly find below the comments raised by my review.

1- In the abstract "abnormal labor progression" would be replaced by abnormality of labor progression.
2- In the abstract "We analyzed the incidence of prolonged second stage of labor and the association between biparietal diameter measured <7 days before delivery and prolonged second stage of labor" would be replaced by we analyzed the incidence of prolonged second stage of labor and its association with fetal biparietal diameter measured <7 days before delivery.
3- In the introduction, line 47, operator assists the delivery can be removed.
4- In the introduction, line 50, what do you mean by " after <37 weeks"? it is either after 37 weeks or before 37 weeks.
5- In the methods, lines 91-96, no need to mention indications for elective or emergency CS.
6- in the results, no data about fetal head position in women with prolonged second stage of labor. This would be considered as a major limitation.
7- Subgroup analysis for nulliparous and multiparous women would be recommended, in table 1 and 2.
8- The duration of the second stage of labor would be examined by survival methods. Any intervention as CS or instrumental delivery would affect the duration of labor.
9- Data about number of patients who required instrumental delivery or CS for arrest of the second stage of labor should be provided.
10- Data about episiotomy need and perineal tears would be provided.
