# Supplementary material for: Fetal biparietal diameter as a potential risk factor for prolonged second stage of labor: A retrospective observational cohort study
Source: PLoS One. 2020 Oct 1;15(10):e0239045. doi: 10.1371/journal.pone.0239045 (PMC7529230; doi:10.1371/journal.pone.0239045)
Supplement: Attachment — Submitted filename: reviewer comments 2.docx [file pone.0239045.s003.docx]

Dear editor,

Many thanks for giving me this opportunity to review this attractive article. The authors have addressed the previous concerns adequately. Kindly find below the comments raised by my review.

1- In the abstract, line 38- 42. This is hard to read. It needs clarification.
2- Line 42, in the contrary, in multiparous women…., there was no mention for nulliparous women at all.
3- Line 134, To identify the potential indicators of prolonged second stage of labor… could be removed and start the sentence with prolonged second stage of labor was defined as……
4- Table 2 would be subdivided into nulliparous and multiparous women so that tables 3 and 6 would be presented in one table (table 2).
5- line 334, Nulliparity should be replaced by nulliparous.
